# Draft Genome Sequences of *Listeria monocytogenes*, Isolated from Fresh Leaf Vegetables in Owerri City, Nigeria

**DOI:** 10.1128/genomeA.00354-17

**Published:** 2017-06-01

**Authors:** Ogueri Nwaiwu, Alexandra Moura, Pierre Thouvenot, Catherine Rees, Alexandre Leclercq, Marc Lecuit

**Affiliations:** a University of Nottingham, Sutton Bonington Campus, Loughborough, Leicestershire, United Kingdom; b Institut Pasteur, National Reference Center and WHO Collaborating Center for *Listeria*, Paris, France; c Institut Pasteur, Biology of Infection Unit, Paris, France; d Inserm U1117, Paris, France; e Paris Descartes University, Sorbonne Paris Cité, Institut Imagine, Necker-Enfants Malades University Hospital, Division of Infectious Diseases and Tropical Medicine, APHP, Paris, France

## Abstract

Here, we report the draft genome sequences of three *Listeria monocytogenes* isolates from fresh leaves collected in Nigeria, belonging to sequence types ST5 and ST155 (sublineages SL5 and SL155, respectively).

*Listeria monocytogenes* is a foodborne Gram-positive bacterium that can cause listeriosis, a severe infection in humans and animals. Recent genomic studies have demonstrated the international circulation of *L. monocytogenes*, highlighting the urgent need to monitor strains at the global level (1).

Here, we report the draft genome of three isolates isolated in Owerri City, southeastern Nigeria, from fresh leaves of *Gnetum africanum*, *Gongronema latifolium*, and *Vernonia amygdalina* vegetables (2).

DNA was purified using the DNeasy blood and tissue extraction kit (Qiagen), from 5 mL of liquid culture grown overnight at 35°C in brain heart infusion medium (BD Difco) under aerobic conditions. DNA quantity and purity was assessed using Qubit (Thermo Fisher Scientific). Library preparation was carried out using the Nextera XT DNA sample kit according to the manufacturer's protocol (Illumina). Whole-genome sequencing was performed on an Illumina NextSeq 500 platform using 2 × 150 bp runs (Illumina, San Diego, CA, USA), at an estimated average read coverage of 130× (Table 1). FqCleaner v3.0 was used to eliminate adapter sequences (3), reduce redundant or over-represented reads (4), correct sequencing errors (5), merge overlapping paired reads (6), and discard reads with Phred scores of ≤20. Assemblies were generated with SPAdes v3.1.0 (7), with different k-mer values ranging from 21 to 127. The draft genome sizes varied from 2.93 to 3.06 Mb, with a G+C content of 37.9% (Table 1). Multilocus sequence typing (MLST) and core genome MLST (cgMLST) allelic profiles were performed from the assemblies using the BIGSdb*-Lm* database (http://bigsdb.pasteur.fr/listeria), as described previously (1). Isolates belonged to MLST ST5 and ST155 (8) and cgMLST profiles were defined as new cgMLST types (CT2050 and CT2051) (1). Gene prediction and annotation were performed by the RAST tool (9).

**TABLE 1:** Genome features of the three *L. monocytogenes* isolates reported in this study

Isolate (genotype^a^)	No. of reads	Avg coverage	No. of contigs	*N*_50_ contig length (bp)	Genome size (bp)	% G+C	No. of genes	Accession no.
LmNG1 (L1-SL5-ST5-CT2050)	1,112,112	111	25	412,544	2,968,175	37.86	2,996	FWPO01000001 to FWPO01000025
LmNG2 (L1-SL5-ST5-CT2050)	1,172,506	117	30	237,962	3,060,847	37.86	3,106	FWPR01000001 to FWPR01000030
LmNG3 (L2-SL155-ST155-CT2051)	1,310,101	131	30	510,628	2,933,139	37.86	2,983	FWPS01000001 to FWPS01000030

^a^ The genotype is indicated as a string of phylogenetic lineage (L), sublineage (SL), sequence type (ST), and cgMLST type (CT).

These isolates constitute the first *L. monocytogenes* genome sequences from Nigeria and will contribute to a better knowledge of the diversity of this species in Africa (10).

## Accession number(s). 

The draft assemblies reported here were deposited at DDBJ/EMBL/GenBank under the accession numbers provided in Table 1.
